# Lexical Tones in Mandarin Chinese Infant-Directed Speech: Age-Related Changes in the Second Year of Life

**DOI:** 10.3389/fpsyg.2018.00434

**Published:** 2018-04-04

**Authors:** Mengru Han, Nivja H. de Jong, René Kager

**Affiliations:** ^1^Utrecht Institute of Linguistics (OTS), Utrecht University, Utrecht, Netherlands; ^2^Leiden University Centre for Linguistics (LUCL), Leiden University, Leiden, Netherlands; ^3^Leiden University Graduate School of Teaching (ICLON), Leiden University, Leiden, Netherlands

**Keywords:** infant-directed speech, lexical tone, prosody, Mandarin Chinese, age effect, word learning

## Abstract

Tonal information is essential to early word learning in tone languages. Although numerous studies have investigated the intonational and segmental properties of infant-directed speech (IDS), only a few studies have explored the properties of lexical tones in IDS. These studies mostly focused on the first year of life; thus little is known about how lexical tones in IDS change as children’s vocabulary acquisition accelerates in the second year (Goldfield and Reznick, 1990; Bloom, 2001). The present study examines whether Mandarin Chinese mothers hyperarticulate lexical tones in IDS addressing 18- and 24-month-old children—at which age children are learning words at a rapid speed—vs. adult-directed speech (ADS). Thirty-nine Mandarin Chinese–speaking mothers were tested in a semi-spontaneous picture-book-reading task, in which they told the same story to their child (IDS condition) and to an adult (ADS condition). Results for the F0 measurements (minimum F0, maximum F0, and F0 range) of tone in the speech data revealed a continuum of differences among IDS addressing 18-month-olds, IDS addressing 24-month-olds, and ADS. Lexical tones in IDS addressing 18-month-old children had a higher minimum F0, higher maximum F0, and larger pitch range than lexical tones in ADS. Lexical tones in IDS addressing 24-month-old children showed more similarity to ADS tones with respect to pitch height: there were no differences in minimum F0 and maximum F0 between ADS and IDS. However, F0 range was still larger. These results suggest that lexical tones are generally hyperarticulated in Mandarin Chinese IDS addressing 18- and 24- month-old children despite the change in pitch level over time. Mandarin Chinese mothers hyperarticulate lexical tones in IDS when talking to toddlers and potentially facilitate tone acquisition and word learning.

## Introduction

In tone languages, pitch is employed to differentiate lexical meanings. Consequently, in order to recognize or learn a word, a tone-language-learning infant must develop sensitivity to lexical pitch contours in addition to consonants and vowels; conversely, infants who learn non-tone languages need to pay attention to consonants and vowels but ignore pitch contours at the lexical level. Though a number of studies have looked at infants’ discrimination, recognition, and acquisition of tones (see Singh and Fu, 2016 for a review), only a few studies have examined lexical tones in early language input—i.e., infant-directed speech (IDS). The results drawn from these studies are inconsistent; some suggest that tones in IDS are hypoarticulated, while others show that they are hyperarticulated compared with tones in adult-directed speech (ADS). Moreover, most previous studies have focused on IDS in the first year of life, when perceptual reorganization is taking place (Werker and Tees, 1984); comparatively little is known about how tonal input changes in the second year, when children start to become verbal and gain vocabulary at a rapid speed (Bloom, 2001). As tonal information is crucial to distinguishing word meanings, the current study investigates whether lexical tones in Mandarin Chinese IDS addressing 18- and 24-month-old children are hyperarticulated—and if so, whether the tonal cues change depending on the age of the child.

Infant-directed speech is a speech register caregivers (typically mothers) use when addressing their infants, and as such it is an important type of input in early language acquisition (Soderstrom, 2007; Cristià, 2013). IDS is known to exhibit exaggerated intonation compared with ADS, including higher pitch, a larger pitch range, and greater pitch variation (Fernald and Simon, 1984; Fernald et al., 1989). These types of prosodic modifications are found in IDS in the majority of world languages, including both non-tone languages, such as English and German (Fernald and Simon, 1984; Fernald et al., 1989; Cristià, 2013), and tone languages, such as Mandarin Chinese, Cantonese, and Thai (Grieser and Kuhl, 1988; Kitamura et al., 2001; Xu Rattanasone et al., 2013). Despite the near-universality of exaggerated intonation, the degree of exaggeration may show cross-linguistic or cross-cultural differences. For instance, American English IDS was found to exaggerate prosody more than British English, Japanese, German, French, and Italian IDS (Fernald et al., 1989). In the IDS of tone languages, lexical tone (pitch at the lexical level) interacts with exaggerated intonation (pitch at the intonational level); as a result, the prosodic modifications expressed in tone-language IDS may differ in meaningful ways from those found in non-tone-language IDS. For instance, Kitamura et al. (2001) found that, although Thai IDS exhibited exaggerated intonation compared with Thai ADS, it was less exaggerated than Australian English IDS.

IDS is often claimed to facilitate language acquisition, although conflicting views have been proposed (Gleitman et al., 1984; Soderstrom, 2007). One line of research has shown that, compared with ADS, IDS attracts infants’ attention more effectively. Infants—even newborns—prefer listening to IDS over ADS (Cooper and Aslin, 1990). This listening preference is probably largely attributable to the positive affect of IDS (Singh et al., 2002). Positive affect is a common characteristic of IDS, and one that shares similar prosodic features with exaggerated intonation (Kitamura and Burnham, 1998). When they manipulated affect and speech register in IDS to examine 6-month-old children’s listening preference, Singh et al. (2002) found that higher pitch and greater pitch variation alone did not account for infants’ preference; positive affect was also required.

The robust evidence that infants prefer listening to IDS, however, does not necessarily indicate that such speech carries a particular linguistic function in terms of language learning. Another line of research has been devoted to identifying the well-specified linguistic information encoded by IDS. A number of studies have explored two questions on this topic: First, are the segmental (mainly vocalic) and suprasegmental (tonal) properties of IDS hyperarticulated compared with those of ADS? It may seem, on first blush, that the exaggerated intonation IDS entails vowel hyperarticulation. However, it is also possible that exaggerated intonation provides more variable vowels, and thus poses a learning problem for vowel categorization. Similarly, exaggerated intonation need not naturally result in tone hyperarticulation; on the contrary, it may distort tonal cues at the syllabic level. Second, if the segmental and suprasegmental properties of IDS are indeed hyperarticulated, is this hyperarticulation expressed in a way that may support language acquisition? Previous investigations into this possibility have produced mixed results on the segmental level (vowels and consonants) and few results of any kind on the suprasegmental level (lexical tones).

An example of vowel hyperarticulation was identified by Kuhl et al. (1997), who compared the articulation of three point vowels (/i/, /a/, and /u/) between ADS and IDS addressing 2- to 5-month-old infants in American English, Russian, and Swedish. They analyzed the “vowel triangles” for the three vowels in IDS and ADS; a larger vowel triangle indicated that the vowels were more distinctive from each other. The results showed that in all three languages, mothers expanded the vowel triangles in IDS compared with ADS, suggesting that mothers produced more distinctive vowels in IDS. Similar results have been obtained in other languages, including Taiwanese Mandarin (Liu et al., 2003), French, and Japanese (Dodane and Al-Tamimi, 2007). However, contradictory findings have also been reported. First, vowel hyperarticulation seems to be restricted to point vowels (/i/, /a/, and /u/); when comparing other vowel contrasts such as [i – I] in American English, Cristià and Seidl (2014) did not find these contrasts to be enhanced in IDS. Second, while robust evidence of vowel hyperarticulation exists for multiple languages, other languages seem to show no trace of this phenomenon. For example, vowels in Cantonese IDS toward 3- to 12-month-old infants were not hyperarticulated compared with vowels in Cantonese ADS (Xu Rattanasone et al., 2013). Similarly, a recent study comparing the vowels in natural Japanese IDS addressing 18- to 24-month-old children with the vowels in read Japanese speech found that, although the IDS vowels were more variable, they did not necessarily show more clarity compared with those in ADS (Miyazawa et al., 2017).

The mixed results on vowel hyperarticulation in IDS are only magnified in studies investigating whether IDS supports language acquisition. On the one hand, Song et al. (2010) showed that vowel hyperarticulation in IDS improved word recognition in 19-month-old children. On the other hand, in a perception study on 6- and 7-month-old children, Trainor and Desjardins (2002) found that the exaggerated pitch contours in IDS helped children’s discrimination of vowels, whereas high pitch hampered vowel discrimination. In sum, whether or not vowels in IDS are hyperarticulated—and whether such hyperarticulation, if it exists, helps children’s language acquisition—is still debatable.

A similar debate may be extended to tone hyperarticulation. Hypothetically, the exaggerated intonation of IDS might affect tonal properties in two possible ways. Specifically, lexical tones in IDS may either be hyperarticulated or alternatively distorted (hypoarticulated) due to the exaggerated prosody. Two types of acoustic evidence may indicate tone hyperarticulation in IDS. First, tones’ acoustic cues may be more prominent in IDS as compared with ADS. For example, as fundamental frequency (F0) is the primary cue to tone in Mandarin Chinese (Howie, 1976), tone hyperarticulation can be indicated by a larger F0 range for Tone 2 (mid-rising tone), Tone 3 (low-dipping tone), and Tone 4 (high-falling tone). Tone 1, a high-level tone, may have a higher F0 in IDS than in ADS. Additionally, tone duration, a secondary cue (Blicher et al., 1990), may also be enlarged in IDS for all four tones. Second, enhancement of tonal contrasts is a possible indicator of tone hyperarticulation in IDS. Such enhancement can be measured by comparing the pitch differences between tone pairs in ADS and IDS, or indicated by a larger tone triangle in IDS (e.g., Tang et al., 2017, to review later). To date, only a handful of studies have looked at lexical tones in IDS. Among the few studies that have performed perceptive or acoustic measurements on lexical tones in IDS, conflicting results emerge.

Results from several studies support the distortion prediction. Papoušek and Hwang (1991) found that tone contours in Mandarin Chinese IDS did not correspond to phonologically expected tone contours. In their study, participants were instructed to produce preselected utterances in role-play contexts, imagining the addressee was a child or an adult. The authors speculated that speakers intuitively sacrificed tonal information at the syllabic level in order to accommodate the IDS intonation. Though the study’s results shed light on people’s intuitive prosodic tuning when talking to children, they do not tell us much about tone production in natural IDS, when mothers and children interact directly. In a later study, Kitamura et al. (2001) collected IDS data from Thai speakers in a more natural setting. Specifically, the researchers recorded the spontaneous speech of mothers interacting with their children naturally at home, every 3 months, from birth until the infants were 12 months old (IDS condition); they also recorded the same participants interacting with adults (ADS condition). They then asked trained Thai phonologists to judge whether the tones in utterance-initial and utterance-final positions remained identifiable. The results showed that tones were slightly less identifiable in Thai IDS than ADS, especially in utterance-final positions.

While these studies suggest that tones may be distorted in IDS compared with ADS, there is also evidence that mothers hyperarticulate tones in IDS. Following the methods in Kuhl et al. (1997), Liu et al. (2007) investigated whether vowel hyperarticulation applied to tones in Taiwanese Mandarin. They performed an acoustic analysis on four Taiwanese Mandarin tones in speech directed at 10- to 12-month-old children. Their stimuli consisted of 12 disyllabic words in which the first syllable (target syllable) varied from Tones 1 to 4 and the second syllable remained Tone 1. In the IDS condition, mothers and their infants played together with pictures or objects corresponding to these stimuli; in the ADS condition, the same mothers talked to an experimenter about the children’s interests in these target words. Mean F0, F0 range and duration of vowels of the target syllables were compared between the two conditions. The results showed that Taiwanese Mandarin tones produced in IDS had a raised mean F0, enlarged F0 range and lengthened duration—suggesting that mothers tended to hyperarticulate tones when speaking to their infants.

Two studies further tested tone hyperarticulation in Cantonese IDS with different measurements. Xu Rattanasone et al. (2013) investigated Cantonese tones in the speech of mothers talking to their 3-, 6-, 9-, and 12-month-old children. The stimuli consisted of three of the six tones in the Cantonese tone inventory: tones 55, 25, and 21. The authors adopted a tone triangle measure from Barry and Blamey (2004). For each tone, F0 values were measured at the point of maximal vowel amplitude and at 50% of the maximum amplitude. These two values were plotted for three tones, making a tone triangle. Similar to the vowel triangles in Kuhl et al. (1997), a larger tone triangle indicated more distinctive tonal contrasts. The results showed that tone triangles were larger in IDS than ADS at 3, 6, and 9 months, indicating tone hyperarticulation for these age groups. However, the observed hyperarticulation was reduced for 12-month-olds, indicating that tones in speech to infants are more distinctive until children reached 12 months of age, at which point tones in ADS and IDS become similar. Significantly, the larger tone triangle found for 3-, 6-, and 9-month-olds mainly stemmed from differences between the high-level tone (55) and the low-level tone (21) (Xu, 2008, p. 111); thus, it remains unknown whether these larger tone triangles indicate tone hyperarticulation across the whole tone inventory. In a recent study, Wong and Ng (2017) examined Cantonese tone hyperarticulation in IDS (toward 7- to 12-month-old infants), using both native judgment and acoustic analysis. They found that tones in Cantonese IDS had higher F0 and longer duration than tones in ADS, but such differences did not seem to facilitate adults’ perception of tonal contrasts. Using the tone triangle measure in Xu Rattanasone et al. (2013), Tang et al. (2017) examined tone hyperarticulation in Northern Mandarin. Interestingly, they only found tone hyperarticulation (for both tone space and duration) when the target tones were in utterance-final position.

Taken together, these findings indicate that Cantonese tones are hyperarticulated in early IDS compared with ADS, but that the degree of hyperarticulation diminishes by the end of the first year. They also suggest that tone hyperarticulation may be restricted to certain tones or positions (as in Northern Mandarin, where tone hyperarticulation is only present in utterance-final positions). In other words, it has not been conclusively established that lexical tones in IDS are hyperarticulated across the board. To date, studies of IDS have been conducted on different languages, with different data collection methods and different measurements, and have yielded conflicting results. These methodological issues must be taken into consideration before we draw any conclusions about the hyperarticulation of tone in IDS.

Tone languages studied in the existing literature on IDS include Cantonese, Mandarin Chinese, Northern Mandarin, and Taiwanese Mandarin, all of which have different tonal systems and prosodic patterns (e.g., Chen et al., 2009). It is certainly possible that the interaction of tone and prosodic modifications in IDS may show cross-linguistic differences. In fact, even among variants of the same language, the characteristics of IDS can differ; for example, as noted above, American English IDS tends to be more exaggerated than British English IDS (Fernald et al., 1989)—certainly implying that languages with different tonal systems or different dialects may differ significantly.

Second, speech elicitation methods used in previous studies range from reading tasks to spontaneous speech, and from home settings to laboratory settings. Papoušek and Hwang (1991) used scripted speech, while Liu et al. (2007) selected target words to elicit speech during mother–child interaction (semi-spontaneous), and Kitamura et al. (2001) collected spontaneous speech data in natural interactions at home. Prosody tends to differ in read speech vs. (semi-)spontaneous speech (De Ruiter, 2015). In spontaneous speech (elicited during “natural mother–child interaction”), the speech context varies according to the activity that is taking place—for example, reading books, playing with toys, or changing diapers. Furthermore, in typical experimental settings, the speech contexts for ADS and IDS conditions are rather different from each other. It is not surprising, then, that IDS may be more distinct from ADS in certain contexts, and less distinct in other contexts. Given this degree of variability, it’s not clear whether the large differences between ADS and IDS reported in certain previous studies may actually have been due to the very different settings and activities in the two conditions.

Finally, previous studies have employed a wide range of analyses to compare the ADS and IDS conditions. Kitamura et al. (2001) used native judgment, whereas other researchers performed acoustic analyses; among the studies that conducted acoustic analyses, different measurements were used. These methodological differences further complicate the task of determining whether or not lexical tones are hyperarticulated in IDS.

Besides the methodological issues discussed above, the different ages of the children in the various studies may also have contributed to the contradictory results. Studies on vowel and tone hyperarticulation to date have mostly focused on IDS directed at children in the first year of life, and these results have often been interpreted from the perspective of “perceptual reorganization” (Werker and Tees, 1984). There is robust evidence showing that infants undergo perceptual reorganization, during the first 12 months of life, as their perception of phonetic categories shifts from language-universal to language-specific. This shift is reflected in infants’ progressively better discrimination of native contrasts and poorer discrimination of non-native contrasts. Such perceptual reorganization develops for consonants, vowels, and lexical tones. Mandarin-learning infants, for instance, show improvement in their discrimination of lexical tones between 6 and 9 months of age, while infants who are learning a non-tone language (e.g., English and Dutch) show a decline in their ability to discriminate tonal contrasts over the same age range (Mattock and Burnham, 2006; Liu and Kager, 2014). Thus, findings on tone hyperarticulation during infancy are usually interpreted as evidence for the facilitating effects of IDS on tone perception: as infants’ speech perception becomes progressively tuned to their native (tonal) language, tone hyperarticulation becomes less prominent. Xu (2008, p. 99), for instance, pointed out that her findings—which indicate that tone hyperarticulation declines at 12 months—are consistent with perceptual reorganization research. However, during the same period of perceptual reorganization, children also start to acquire words. Infants start to show recognition of common words as early as 6–9 months (Bergelson and Swingley, 2012), and usually utter their first words around their first birthday. In the second year of life, both receptive and productive vocabulary accelerate at an astonishing speed (Goldfield and Reznick, 1990; Bloom, 2001).

Since tonal information is crucial to word meaning in tone languages, it is important to examine whether tone hyperarticulation persists when children are becoming proficient word-learners in the second year. The general prosodic modifications in IDS are known to change based on the child’s stage of language development (Stern et al., 1983; Kitamura et al., 2001). In general, IDS becomes more ADS-like as children grow older. Taking the perspective of word learning, tone hyperarticulation may not stop when children are 1 year old; on the contrary, it may persist, aiding children’s lexical development as they move into the word-learning phase. As most studies to date have focused on the first year of life, little is known about whether tone hyperarticulation remains present in the second year. Consequently, the timeline of age-related changes in tone hyperarticulation is not well-described in the literature.

Two studies have investigated age-related changes in lexical tones in IDS, but both focused on the first year of life, prior to the lexical spurt. Kitamura et al. (2001) showed that lexical tones in Thai were distorted in IDS directed at children up to 9 months old, but that IDS directed at 12-month-old children did not differ significantly from ADS in tone identification. Results from Xu Rattanasone et al. (2013) showed similar age-related changes: Cantonese tones were hyperarticulated in IDS compared to ADS until 12 months of age, at which point this hyperarticulation was reduced. The authors interpreted their results as evidence that mothers modify their speech according to children’s stages of language development. As infants tune their tone perception toward their native language in the first year of life (Mattock and Burnham, 2006; Mattock et al., 2008; Yeung et al., 2013), tone hyperarticulation declines accordingly.

If age-related changes in IDS are explicitly tied to perceptual reorganization, we should expect any differences between ADS and IDS to diminish and disappear altogether as children reach 12 months of age. However, in a longitudinal study, Liu et al. (2009) found that speech directed to 5-year-old children still showed both general prosodic exaggeration and tone hyperarticulation compared with ADS, though it was less exaggerated than IDS directed at preverbal children. But what happens to IDS directed at children between infancy (up to 12 months) and school-age (5 years old)? There is a gap in the existing investigations of tone hyperarticulation during this period. The present study seeks to fill that gap by asking what happens to tones in IDS in the second year of life, when children start to talk and learn vocabulary at a high rate. It remains an open question whether mothers speaking tone languages alter their tones in IDS to facilitate tone acquisition (and, consequently, lexical development) in their children. If tone hyperarticulation is not restricted to supporting perceptual reorganization, we should find evidence for tone hyperarticulation in IDS addressing 18- and 24-month-olds.

The current study set out to investigate tone hyperarticulation in Mandarin Chinese IDS at two points in time, both of which occur during the second year of life (the period of the lexical spurt). Our main research questions are: (1) Are tones in Mandarin IDS addressed to 18- and 24-month-old children generally hyperarticulated compared to tones in ADS? If so, we should expect to observe a larger F0 range for Tone 2, Tone 3, and Tone 4, a higher F0 for Tone 1, and possibly longer duration for tones in IDS vs. ADS, as shown by Liu et al. (2007). In addition to these general measures, we explored whether lexical tonal contrast between Tones 1 and 4 was enhanced in IDS. (2) Do lexical tones in Mandarin Chinese IDS change when the mother is addressing an 18-month-old child vs. a 24-month-old child? We predict that as children’s vocabulary size increases significantly from 18 to 24 months, the lexical tonal cues change. Specifically, tonal cues in IDS should be more similar to ADS when children reach 24 months’ old. To address these questions, we collected speech samples from a story-telling task, where mothers told a story containing target words featuring four Mandarin Chinese tones to their 18- and 24-month-old children (IDS condition), and to an adult control (ADS condition).

## Materials and Methods

### Participants

Thirty-nine Mandarin-Chinese-speaking mother–child dyads participated in this study. The participant sample comprised two age groups: 18-month-olds (*N* = 21; mean age = 18;15; age range = 17;21 – 18;27; girls *N* = 9) and 24-month-olds (*N* = 18; mean age = 24;15; age range = 23;27– 24;27; girls *N* = 10). All participants were recruited from kindergartens in Yichang, China. All the participant mothers spoke Mandarin Chinese^1^ (the official language in China), as well as a dialect (in this case, Southwest Mandarin). The participant children heard this dialect in their language community, but were exposed to Mandarin Chinese at home, at kindergarten, and in the national media. This type of bilingual language background is common for most people in China (Li and Lee, 2006). To obtain a homogeneous group of participants, we set these criteria in our recruiting interview: (1) the mothers should speak Mandarin Chinese with good proficiency; (2) the mothers should mostly speak Mandarin Chinese to their children at home; and (3) the children should be learning Mandarin Chinese as one of their first languages.

### Materials

A picture book titled *Xiaotuzi de yitian* (“Bunny’s day”) was designed to elicit four target words for 18- and 24-month-old children (see **Table 1**). On each page of the book, one word appeared on the left side, and a corresponding picture appeared on the right side. The pages contained no text beyond these target words. An additional six pages were used as fillers and to make the story coherent. The target words were all disyllabic nouns, of which the first syllable was always Tone 2 (a rising tone), and the second syllables varied from Tones 1 to 4. We chose Tone 2 for the first syllable in order to ensure consistent tonal coarticulation effects (i.e., carry-over effects on the following tone) across tokens and registers.

**Table 1:** Overview of stimuli.

Tone of the second syllables	Tone 1	Tone 2	Tone 3	Tone 4
Pinyin	nán guā	hé lí	chéng bǎo	mí lù
IPA	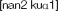	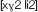	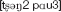	[mi2 lu4]
Translation	‘Pumpkin’	‘Beaver’	‘Castle’	‘Moose’

### Procedure

Participants were tested in a quiet room. Before the experiment, mothers were given a few minutes to get familiar with the book. In the IDS condition, the child sat on his or her mother’s lap, and the mother was instructed to read the story to her child the way she usually did at home. The mothers were specifically told they could use any sentences; the only requirement was to include the words on each page. In the ADS condition, the mothers were instructed to tell the story to the experimenter (female, a native speaker of Mandarin Chinese), taking into account that she was a college student. This was done to control the speech context and content in both conditions. The order of the two conditions was counterbalanced across participants. A ZOOM H1 recorder (with 16-bit resolution and a sampling rate of 44.1 kHz) was used to make audio recordings, and all sessions were videotaped. Each experimental session took about 15–20 min. All families received a book as a gift after the session.

## Data Analysis and Results

### Data Analysis

The beginnings and endings of the target syllables (the second syllable of each target word) were annotated and extracted from the recordings in PRAAT (Boersma and Weenink, 2017), following the phonetic segmentation principles in Skarnitzl and Machač (2011). In total, 713 target syllables were extracted; of these, 47 syllables (6.6%) were excluded due to background noise or interference from a child’s voice.

We chose to acquire the maximum and minimum F0 for each syllable by marking them manually, rather than limiting tone measures to any specific segment(s) within the syllables. This was done for two reasons. First, the domain of tones (or Tone Bearing Units (TBUs)) is phonologically determined, and what constitutes a TBU in Mandarin Chinese is debatable (see Zhang, 2014, p. 81 for a review). Phonetic studies have shown that the voiced parts of syllables—i.e., vowels, initial voiced consonants, prenuclear onglides, and nasal codas—may convey tonal information (Howie, 1974; Duanmu, 2007). In studies involving acoustic analyses of lexical tones in IDS, however, the common practice has been to identify tones based on the F0 measures on vowels, potentially leading to the exclusion of other segments that may carry pitch contours. Second, contextual tonal variations in natural speech—for example, anticipatory and carry-over effects in adjacent Mandarin tones (Xu, 1997)—may also make it difficult to extract pitch measures accurately using an automatic method. In previous studies, the stimuli were either monosyllabic (Xu Rattanasone et al., 2013) or associated with the first syllable of the target words in natural speech, where the carry-over effects from the pre-target syllables were uncertain (Liu et al., 2007). Such methods disregard the potential for contextual impact from adjacent tones. In the current study, we made sure that the first syllable of the target words was always Tone 2 (a rising tone), so that the first syllable had a similar effect on the second tone for each target word.

Taking these issues into account, to get a more accurate picture of tonal information, the first author manually marked the maximum F0 and minimum F0 following the methods from Chen and Gussenhoven (2008). As a secondary cue to tones, durations of syllables were extracted automatically using a Praat script (Lennes, 2017). Using these techniques, we obtained four dependent measures for each target syllable: Minimum F0, Maximum F0, F0 range (Maximum F0 – Minimum F0), and Duration of syllables (in seconds). Tone 1 was excluded in the F0 range analyses since it is a flat tone, for which the pitch height (not the pitch range) is the major cue.

For all the F0 measures, we followed Liu et al. (2007) and used two scales: (1) Hz, a linear pitch scale that has been used traditionally in phonetic research; and (2) Equivalent-rectangular-bandwidth-rate (ERB), which has been found to better describe pitch perception (Hermes and van Gestel, 1991).

### Results

#### General Tone Hyperarticulation

To understand whether tones differed between (i) ADS and IDS and (ii) IDS directed at 18-month-olds and IDS directed at 24-month-olds, we used linear mixed-effects models for all analyses. In the models, we included fixed factors of Age (18-month-old/24-month-old), Condition (ADS/IDS) and Tone (Tones 1, 2, 3, and 4) on these dependent measures: Minimum F0 (in Hz and ERB), Maximum F0 (in Hz and ERB), F0 range (in Hz and ERB, for Tone 2, Tone 3 and Tone 4, excluding Tone 1), and Syllable duration (in seconds), with Participant Number as a random factor, and allowing for random slopes for Condition and Tone (Barr et al., 2013). All dependent measures were square-root transformed from raw data to get a more normalized distribution (indicated by *W* in Shapiro–Wilk test).

We used the lme4 package (Bates et al., 2017) in the R environment (R Development Core Team, 2016) for all data analyses. For each dependent measure, we took the backward elimination approach, starting with a model that included all fixed effects plus the random factor, and all interactions between them (the most complex model)^2^ (Bates et al., 2015a). Then, we used the “step” function in the lmerTest package (Bates et al., 2015b, p. 15) to reduce the models by eliminating non-significant factors or interactions. When we arrived at an interaction of the fixed effects Condition and Age in the final models, we split the data by Age and built further models for each age group^3^. For Maximum F0 (Hz) and Minimum F0 (ERB), the models with maximal random effects failed to converge. Therefore, we excluded Tone as a random effect^4^ for these two measures. As the results were consistent across Hz and ERB for all pitch measures, we only present results in Hz here. The results of F0 measures in ERB can be found in Supplementary Material. In the following subsections, we report on the final models for each dependent measure. Our main aim was to investigate the general tone hyperarticulation phenomenon in Mandarin Chinese IDS; hence we focus on the fixed effects of Condition and Age, as well as the interaction between these two factors. To further explore whether tonal contrasts are enhanced in IDS, we present an exploratory analysis on the enhancement of Tone 1 – Tone 4 contrast in Section “Exploring the Enhancement of Tone 1 – Tone 4 Contrast.”

**FIGURE 1:**
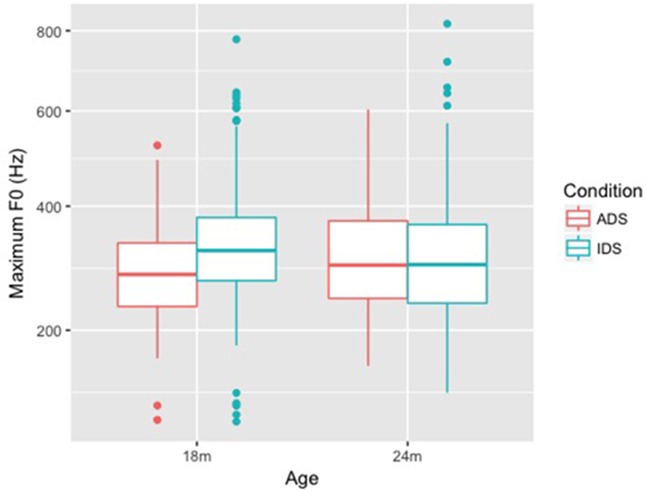
Box plots of Maximum F0 (Hz) for ADS and IDS addressing 18- and 24-month-old children^5^ .

For Maximum F0 (Hz) (**Figure 1**), the final model (**Table 2**) revealed a significant main effect of Condition (*p* = 0.001), as well as a significant interaction of Condition and Age (*p* = 0.015). To further examine the different effects of Condition on Maximum F0 in the two age groups, we split the data by Age. The models for the two age groups showed a significant main effect of Condition for the 18-month group (β = 1.401, *SE* = 0.398, *t* = 3.516, *p* = 0.002), but not for the 24-month group, suggesting that there was no effect of Condition on Maximum F0 for IDS directed at 24-month-olds. The final models for Maximum F0 and Minimum F0 for each age group can be found in Supplementary Material. Thus, the Maximum F0 of lexical tones was higher in IDS than in ADS only in the 18-month-old group. By the time children were 24 months old, there was no difference between the two speech registers with respect to Maximum F0.

**Table 2:** Final model for Maximum F0 (Hz).

Parameters	Estimate	*SE*	*t*-value	*p*
**Fixed factors**			
(Intercept)	16.8043	0.401	41.942	<0.001***
Condition (IDS)	1.403	0.400	3.518	0.001**
Tone2	-0.483	0.259	-1.866	0.063
Tone3	-0.893	0.253	-3.532	<0.001***
Tone4	1.130	0.246	4.600	<0.001***
Age (24 months)	0.647	0.538	1.202	0.237
Condition (IDS): Age (24 months)	-1.480	0.582	-2.545	0.015*

*^∗^p < 0.05, ^∗∗^p < 0.01, ^∗∗∗^p < 0.001.*

**FIGURE 2:**
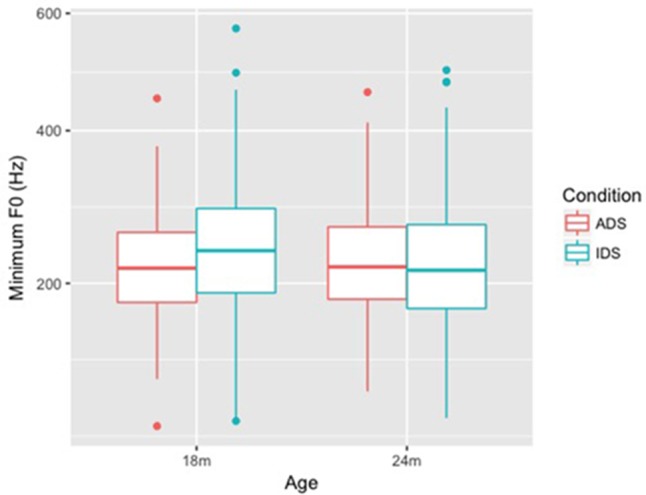
Box plots of Minimum F0 (Hz) for ADS and IDS addressing 18- and 24-month-old children.

Results for Minimum F0 (Hz) (**Figure 2**) showed a similar pattern: the final model (**Table 3**) showed a significant main effect of Condition (*p* = 0.030) and a significant interaction of Condition and Age (*p* = 0.014). When we split the data by Age, we found that there was a significant main effect of Condition for the 18-month-old group (β = 0.589, *SE* = 0.224, *t* = 2.630, *p* = 0.010), but not for the 24-month-old group, as Condition was not in the final model. The results reveal that, similar to Maximum F0, Minimum F0 was also significantly higher in IDS addressing 18-month-old children than in ADS, while no similar differences in Minimum F0 arose between ADS and IDS for the 24-month-old group.

**Table 3:** Final model for Minimum F0 (Hz).

Parameters	Estimate	*SE*	*t*-value	*p*
**Fixed factors**			
(Intercept)	16.214	0.334	48.600	<0.001***
Condition (IDS)	0.552	0.248	2.225	0.030*
Tone2	-1.981	0.300	-6.608	<0.001***
Tone3	-2.970	0.274	-10.825	<0.001***
Tone4	-1.161	0.252	-4.610	<0.001***
Age (24 months)	0.390	0.408	0.954	0.346
Condition (IDS): Age (24 months)	-0.870	0.352	-2.548	0.014*

*^∗^p < 0.05, ^∗∗^p <0.01, ^∗∗∗^p < 0.001.*

**FIGURE 3:**
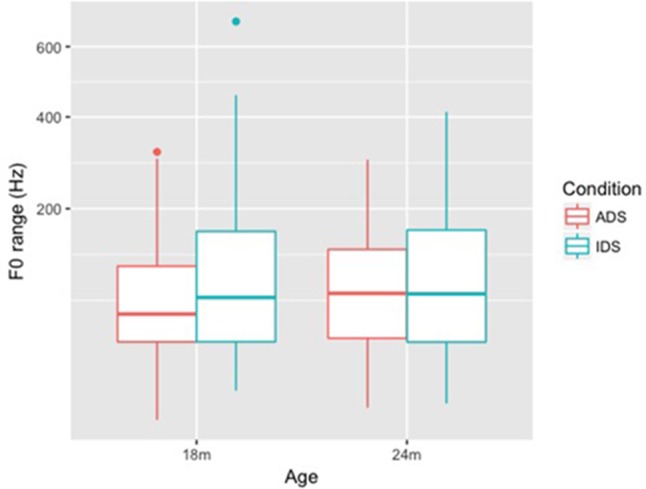
Box plots of F0 range (Hz) for ADS and IDS addressing 18- and 24-month-old children.

For the measure of F0 range (**Figure 3**), the final model (**Table 4**) only showed a significant main effect of Condition (*p* = 0.006); no interaction between Age and Condition was observed on this measure, suggesting that lexical tones in Mandarin Chinese IDS have a larger F0 range than ADS tones across the two age groups.

**Table 4:** Final model for F0 range (Hz).

Parameters	Estimate	*SE*	*t*-value	*p*
**Fixed factors**				
(Intercept)	8.201	0.576	14.227	<0.001***
Condition (IDS)	0.969	0.352	2.751	0.006*
Tone3	0.050	0.632	0.079	0.937
Tone4	0.480	0.600	0.800	0.424
Age (24m)	-1.288	0.764	-1.686	0.094
Tone3: Age (24 months)	1.848	0.884	2.091	0.037*
Tone4: Age (24 months)	2.467	0.855	2.884	0.004**

*^∗^p < 0.05, ^∗∗^p < 0.01, ^∗∗∗^p < 0.001.*

**FIGURE 4:**
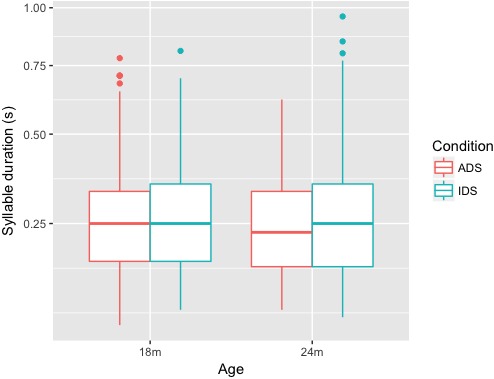
Box plots of syllable duration (s) for ADS and IDS addressing 18- and 24-month-old children.

The last measure was duration (**Figure 4**). For this measure, the final model (**Table 5**) did not include Condition, suggesting that there was no effect of Condition on duration for either the 18-month-old or the 24-month-old groups.

**Table 5:** Final model for syllable duration (s).

Parameters	Estimate	*SE*	*t*-value	*p*
**Fixed factors**				
(Intercept)	0.493	0.011	44.314	<0.001***
Tone2	0.070	0.014	5.230	<0.001***
Tone3	-0.003	0.015	-0.197	0.844
Tone4	0.028	0.013	2.137	0.034*

*^∗^p < 0.05, ^∗∗^p < 0.01, ^∗∗∗^p < 0.001.*

#### Exploring the Enhancement of Tone 1 – Tone 4 Contrast

Our main goal was to provide a global measure of tone hyperarticulation, however, tone hyperarticulation may also suggest that tonal contrasts are enhanced in IDS. In addition to comparing the tonal cues between ADS and IDS, we explored whether the contrast between Tone 1 and Tone 4 was enhanced in IDS^6^. Both the contrast between Tone 1 (high-level tone) and Tone 4 (high-falling tone) and the contrast between Tone 2 (mid-rising tone) and Tone 3 (low-dipping tone) are typically used in studies on infant tone perception (e.g., Chen et al., 2017; Liu and Kager, 2017). As the realization of Tone 3 has a large degree of variation in spontaneous speech depending on various factors (e.g., Tone 3 sandhi and the position of a Tone 3 syllable in an utterance, see Yip, 2002), it is impossible to gage the enhancement of Tones 2 – 3 contrast from the current data. Thus, we opted instead to focus on the tonal contrast between Tones 1 and 4 and explored whether this tonal contrast was enhanced in IDS as compared with ADS. Since Tones 1 and 4 are mainly distinguished by pitch range, if the difference in pitch range between Tones 1 and 4 was larger in IDS than in ADS, we can conclude that the contrast between Tones 1 and 4 was enhanced in IDS.

First, we took all occurrences of Tones 1 and 4 across the two age groups into analysis. A paired samples *t*-test showed that there was a marginally significant difference (*t* = -2.024, df = 35, *p* = 0.051) in the difference in pitch range (Tones 4 – 1) between IDS (mean = 97.724 Hz, *sd* = 75.934) and ADS (mean = 66.987 Hz, *sd* = 60.927). As in Liu et al. (2007), we then further considered the first two occurrences of each tone. A paired samples *t*-test showed that there was a significant difference (*t* = -2.294, df = 31, *p* = 0.029) in the difference in pitch range (Tones 4 – 1) between the two conditions (ADS: mean = 65.408 Hz, *sd* = 55.525; IDS: mean = 103.397 Hz, *sd* = 88.669) as compared with ADS. Taken together these results showed that the contrast between Tones 1 and 4 was enhanced in IDS, especially for the first two occurrences of the target syllables.

#### Results Summary

Both Minimum F0 and Maximum F0 of lexical tones were higher (in both Hz and ERB) in IDS addressing 18-month-old children than in ADS, but no similar differences were observed between ADS and IDS addressing 24-month-children. This pattern suggests that mothers in the study raised the pitch level of tones when they addressed 18-month-old children, but maintained ADS-like pitch height when addressing 24-month-olds. F0 range (Hz and ERB), on the other hand, showed a difference between ADS and IDS across ages: F0 range was larger in IDS compared with ADS for both 18- and 24-month-olds. As for duration, our results showed that tones were not lengthened in either age group.

Our results showed that tone hyperarticulation was present in IDS addressing 18- and 24-month-old children, but the specific tonal cues differed between the two groups: for 18-month-olds, Tone 1 had a higher F0 in IDS, and Tones 2, 3, and 4 had higher F0 and a larger F0 range in IDS. For 24-month-olds, all four tones remained the same pitch level in the two speech registers, though Tones 2, 3, and 4 in IDS still had a larger pitch range in IDS. As a secondary cue to lexical tone (Blicher et al., 1990), duration did not differ between ADS and IDS in either age group. In addition, an exploratory analysis showed that the contrast between Tones 1 and 4 was enhanced in IDS.

## Discussion and Conclusion

This study examined lexical tones in Mandarin Chinese IDS addressing 18- and 24-month-old children, at the age of the vocabulary spurt. The study had two main goals: to test whether tones are hyperarticulated in IDS compared with ADS in Mandarin Chinese, and to explore how tones in IDS vary with the age of the addressee during the period of vocabulary spurt. To accomplish these goals, we measured the acoustic cues of lexical tones in ADS and IDS in a semi-spontaneous story-telling task. The results demonstrated that tone hyperarticulation and age-related changes are observed in Mandarin Chinese IDS addressing toddlers.

Our research questions were: (i) Are tones in Mandarin IDS addressed to 18- and 24-month-old children hyperarticulated compared to tones in ADS? (ii) Do lexical tones in Mandarin Chinese IDS change when the mother is addressing an 18-month-old child vs. a 24-month-old child? Our results build on past studies on lexical tones in IDS addressing preverbal children (Liu et al., 2007; Xu Rattanasone et al., 2013), while extending that research to the second year of life—the period of the vocabulary spurt. Our findings show that tone hyperarticulation remains present in speech to toddlers, even after their phonetic perception has tuned to their native language and they have started learning words. Specifically, we found that, in speech addressed to 18-month-old children, both the minimum and maximum F0 of tones was higher in IDS than ADS, and the F0 range was larger, but the tones were not lengthened. These F0 measures are consistent with the findings of Liu et al. (2007) for IDS addressing 12-month-old children. In speech addressed to 24-month-old children, we found that pitch height of lexical tones had normalized to the ADS standard, while F0 range remained larger in IDS than ADS. Tone duration does not appear to differ between toddler-addressed IDS and ADS.

Taken in the context of previous studies exploring lexical tones in IDS addressing preverbal children and preschool children, our results contribute to the timeline of tonal changes in IDS by providing evidence for tone hyperarticulation in the second year. Xu Rattanasone et al. (2013) demonstrated that tones in Cantonese IDS are hyperarticulated when talking to children from 3 to 9 months, but that hyperarticulation declines as children approach 12 months. In their study on Taiwanese Mandarin, Liu et al. (2007) found that when addressing 12-month-old children, mothers exaggerated every acoustic correlate of tone in IDS, including producing a higher F0, larger F0 range, and longer duration. The current findings fill a crucial gap in the timeline and suggest that tone hyperarticulation may continue until children reach their second birthday. Liu et al. (2009) compared tones in Taiwanese Mandarin–speaking mothers’ speech to preverbal children (IDS, age range: 0;7–1;0) and speech to preschool children (CDS: age: 5;0), and found that the degree of tone exaggeration was much less in CDS than IDS. Based on this evidence, we may tentatively trace a developmental trajectory of tone hyperarticulation in IDS: hyperarticulation is notably salient from birth to 12 months in both F0 and duration measures, remains present for F0 measures of tone at 18 months, but begins normalizing toward the ADS standard by the end of the second year. By 24 months, the degree of pitch height difference between ADS and IDS drops significantly for all four tones, although pitch range (of Tones 2, 3, and 4) remains larger in IDS compared with ADS. However, simply combining these findings is not sufficient to produce a complete picture of the developmental trajectory of how lexical tones change with age in IDS, since the studies noted above investigated different tone languages, and adopted different acoustic measures and different elicitation methods.

A question that follows from these findings is: why do tonal cues in IDS change over time? It seems likely that the change in the pitch level (minimum and maximum F0) is related to the general prosodic exaggeration, as the degree of prosodic exaggeration in IDS may also decline from 18 to 24 months when children have become more verbal and their word learning accelerates. However, since studies on tone hyperarticulation (including the current study) usually focus on the syllabic level, little is known about whether tonal cues coincide with other prosodic features of IDS. Crucially, our results showed that the pitch range (of Tones 2, 3, and 4) remained enlarged in IDS even when the pitch height had declined to the ADS level at 24 months, suggesting that mothers may hyperaticulate lexical tones during the period of vocabulary spurt in support of word learning.

A relationship between the quality of IDS and children’s language development has often been assumed in research on IDS (e.g., Fernald and Simon, 1984), and the hyperarticulation phenomenon has been offered up as evidence for the facilitative effects of IDS on language acquisition. However, although phonetic input is clearly exaggerated in IDS, at the same time, it is also highly variable compared with the input observed in ADS (Adriaans and Swingley, 2017). Might this variability make it *more* difficult for infants to form phonetic categories? Adriaans and Swingley’s (2017) research suggests not: the authors used categorization models to train two datasets of hyperarticulated vowels (IDS-characterized) and non-exaggerated vowels (ADS-characterized), and found that the highly variable vowels in IDS favored phonetic categorization compared with the non-exaggerated vowels. However, empirical research on whether IDS indeed supports language acquisition—and more specifically, tone acquisition and word learning in tone languages—is surprisingly lacking. Future research should examine whether raised pitch and/or enlarged pitch range indeed facilitates children’s word recognition and word learning.

Thus, we must be cautious in interpreting our results as direct evidence for the linguistic function of IDS in word learning. Indeed, although the current study demonstrates that tone hyperarticulation remains present in language input during the vocabulary spurt period, it does not necessarily indicate that children benefit from this linguistic phenomenon. Several studies have explored the correlation between the quality of IDS and children’s language outcomes. For instance, Liu et al. (2003) found that the vowel space in Taiwanese Mandarin IDS toward preverbal children (6–8 months; 10–12 months) is related to infants’ performance on speech discrimination. Hartman et al. (2017) further showed that the quality of vowels in early English IDS may predict vocabulary size among 2-year-olds. In a word-learning study, Ma et al. (2011) found that 21-month-old English-learning children could only learn words in the IDS condition, while 27-month-old children could learn words successfully in both IDS and ADS conditions. For tone languages, the correlated question—whether tone hyperarticulation in IDS indeed benefits lexical word learning—remains under-investigated. At this point, no research exists directly comparing word learning under ADS and IDS conditions in tone languages, and the literature offers no insight into how tones in language input correlate with vocabulary outcomes.

The pitch measures of tones addressed to our 24-month-old group showed a different pattern from the findings in Liu et al. (2007). In addition to the different age groups under investigation, an alternative explanation for the inconsistent results may be attributed to language-specific properties. Even though Mandarin Chinese (spoken in mainland China) and Taiwanese Mandarin are variations of the same language, their sentential prosody differs (Chen et al., 2009), which may in turn affect the prosody of IDS. Literature comparing the prosody of IDS in Taiwanese Mandarin and Mandarin Chinese is lacking. As British English and American English IDS exhibit different prosodic features (Fernald et al., 1989), one direction invited by the current research is to compare tone hyperarticulation in different tone languages, as well as different variations of the same tone language.

A limitation of the current design is that we used only one target word for each tone. We also took steps to avoid generating contrasts between the target words by ensuring that the phonemes of the target syllables differed from each other. As vowels and tones may interact (Hoole and Hu, 2004), our results are not generalizable to all syllable–tone combinations in Mandarin Chinese. However, it should be noted that this line of research typically relies on a rather small set of stimuli due to the practicalities of testing children. For example, Kuhl et al. (1997) used one target word per vowel; Liu et al. (2007) had twelve syllables for four tones, but they only included “the first two clear tokens of each target word”; Tang et al. (2017) also used one syllable for each tone. Even though there has been some agreement on the salience of tone hyperarticulation in the first year of life, it has not been established that tone hyperarticulation is present across the board. Meta-analysis of existing tone hyperarticulation studies may provide a better understanding of this issue. Also, the current study took a cross-sectional design. We found no effect of Age across ADS and IDS, indicating that there are no group differences between the 18- and 24-month-old groups. However, a timetable of changes in tone hyperarticulation over time remains to be revealed by longitudinal studies.

Another useful future direction for study would be to examine whether tone hyperarticulation is related to the prosodic marking of focused words. Previous research has shown that, in English IDS, mothers tend to put contextually new words (focused words) at utterance-final positions, and these focused words usually carry prosodic marking in the form of higher pitch and a larger pitch range (Fernald and Mazzie, 1991). Relatedly, Tang et al. (2017) showed that tone hyperarticulation in Northern Mandarin only occurs at utterance-final position. In their experimental design, toys corresponding to the target words were provided one by one to each participant (thus, each target word was contextually new). As Mandarin Chinese and English are both SVO languages, it is certainly possible that tone hyperarticulation in Northern Mandarin, as in English, tends to occur when the lexical item in question is the focus of an utterance.

## Conclusion

This study investigated the tone hyperarticulation phenomenon in Mandarin Chinese IDS in the second year of life and revealed age-related changes of tonal cues in IDS addressed to 18-month-old vs. 24-month-old children. These findings may contribute to an understanding of the role of IDS in tone acquisition and word learning. Mothers may hyperarticulate lexical tones in order to provide more fine-grained information for language acquisition. However, it may be premature to interpret these findings as direct evidence for the linguistic function of IDS.

## Ethics Statement

There was no ethical committee in Utrecht Institute of Linguistics (UiL OTS), Utrecht University when data was collected for this study. This study was approved by UiL OTS and was carried out in accordance with the research guidelines in UiL OTS. All participants gave written informed consent.

## Author Contributions

MH contributed to the design of the experiments, data collection, phonetic annotation, data analysis, and drafting the manuscript. NdJ and RK contributed to the experimental design, data analysis, and revision of the manuscript.

## Conflict of Interest Statement

The authors declare that the research was conducted in the absence of any commercial or financial relationships that could be construed as a potential conflict of interest.

## Footnotes

1 We use the term “Mandarin Chinese” in this paper in reference to “Putonghua” or “Standard Chinese,” the official language spoken in China. It should be distinguished from Taiwanese Mandarin, another variety of Mandarin Chinese spoken in Taiwan.

2 An example of the R codes for these models is: sqrt(max_hz) ∼ Condition ^∗^ Tone ^∗^ Age + (1 + Condition + Tone| Participant Number).

3 An example of the R codes is: sqrt(max_hz) ∼ Condition ^∗^ Tone + (1 + Condition + Tone| Participant Number).

4 An example of the R codes is: sqrt(max_hz) ∼ Condition ^∗^ Tone + (1 + Condition| Participant Number).

5 The box plots in our paper show the first and third quantiles, medians, and outliers (included in analysis). All *Y*-axes are square-root transformed.

6 It should be noted that the results reported in Section “Exploring the Enhancement of Tone 1 – Tone 4 Contrast” are exploratory. Our tokens per tone were few resulting in low statistical power for this specific analysis. An alternative way of measuring tonal contrast enhancement would be to compare the tone space area of selected tones (Tones 1, 2, and 4) in ADS and IDS as in Tang et al. (2017). Tang et al. (2017) found that tones in Mandarin Chinese IDS were only hyperarticulated at the utterance-final positions. However, in our current data set, only 18 out of 39 participants had tones (Tones 1, 2, and 4) produced at utterance-final positions in both ADS and IDS conditions, meaning that tone space area could only be compared for these participants. As a result, such an analysis was not feasible for our data set.
