# Supplementary material for: Lexical Tones in Mandarin Chinese Infant-Directed Speech: Age-Related Changes in the Second Year of Life
Source: Front Psychol. 2018 Apr 4;9:434. doi: 10.3389/fpsyg.2018.00434 (PMC5893784; doi:10.3389/fpsyg.2018.00434)
Supplement: Supplementary file 1 [file Table_1.docx]

Supplementary Material

Lexical tones in Mandarin Chinese infant-directed speech: age-related changes in the second year of life

**Mengru Han^1*^, Nivja de Jong^2, 3^, and René Kager^1^**

^1^Utrecht Institute of Linguistics (OTS), Utrecht University, Utrecht, Netherlands

^2^Leiden University Center for Linguistics (LUCL), Leiden University, Leiden, Netherlands

^3^Leiden University Graduate School of Teaching (ICLON), Leiden University, Leiden, Netherlands

***Correspondence:**Mengru Han
han.mengru@gmail.com

# 1. Supplementary Figures and Tables

## Supplementary Tables

**Supplementary Table 1.** **Model for Maximum F0 (Hz) for the 18-month-old group**

| ***Parameters*** | | ***Estimate*** | | ***SE*** | ***t-value*** | ***p*** |
| --- | --- | --- | --- | --- | --- | --- |
|  | *Fixed factors* | |  | |  |  |
| (Intercept) | 16.809 | | 0.390 | | 43.056 | <0.001*** |
| Condition (IDS) | 1.401 | | 0.398 | | 3.516 | 0.002** |
| Tone2 | -0.116 | | 0.342 | | -0.339 | 0.735 |
| Tone3 | -1.097 | | 0.333 | | -3.293 | 0.001** |
| Tone4 | 1.017 | | 0.319 | | 3.191 | 0.002** |

**Supplementary Table 2. Model for Maximum F0 (Hz) for the 24-month-old group**

| ***Parameters*** | | ***Estimate*** | | ***SE*** | ***t-value*** | ***p*** |
| --- | --- | --- | --- | --- | --- | --- |
|  | *Fixed factors* | |  | |  |  |
| (Intercept) | 17.420 | | 0.365 | | 47.710 | <0.001*** |
| Tone2 | -0.872 | | 0.388 | | -2.247 | 0.025* |
| Tone3 | -0.727 | | 0.380 | | -1.914 | 0.057 |
| Tone4 | 1.227 | | 0.375 | | 3.273 | 0.001** |

**Supplementary Table 3. Model for Minimum F0 (Hz) for the 18-month-old group**

| ***Parameters*** | | ***Estimate*** | | ***SE*** | ***t-value*** | ***p*** |
| --- | --- | --- | --- | --- | --- | --- |
|  | *Fixed factors* | |  | |  |  |
| (Intercept) | 16.080 | | 0.325 | | 49.530 | <0.001*** |
| Condition (IDS) | 0.589 | | 0.224 | | 2.630 | 0.010** |
| Tone2 | -1.907 | | 0.313 | | -6.091 | <0.001*** |
| Tone3 | -2.834 | | 0.305 | | -9.283 | <0.001*** |
| Tone4 | -0.834 | | 0.292 | | -2.852 | 0.005* |

**Supplementary Table 4. Model for Minimum F0 (Hz) for the 24-month-old group**

| ***Parameters*** | | ***Estimate*** | | ***SE*** | ***t-value*** | ***p*** |
| --- | --- | --- | --- | --- | --- | --- |
|  | *Fixed factors* | |  | |  |  |
| (Intercept) | 16.282 | | 0.311 | | 52.031 | <0.001*** |
| Tone2 | -1.775 | | 0.321 | | -5.525 | <0.001*** |
| Tone3 | -2.844 | | 0.315 | | -9.037 | <0.001*** |
| Tone4 | -1.292 | | 0.311 | | -4.161 | <0.001*** |

**Supplementary Table 5. Final model for Maximum F0 (ERB) for the 18-month-old and 24-month-old group**

| ***Parameters*** | | ***Estimate*** | | ***SE*** | ***t-value*** | ***p*** |
| --- | --- | --- | --- | --- | --- | --- |
|  | *Fixed factors* | |  | |  |  |
| (Intercept) | 2.629 | | 0.044 | | 59.459 | <0.001*** |
| Condition (IDS) | -0.148 | | 0.043 | | 3.405 | <0.002*** |
| Tone2 | -0.053 | | 0.029 | | -1.865 | 0.063 |
| Tone3 | -0.100 | | 0.028 | | -3.591 | 0.001** |
| Tone4 | 0.125 | | 0.027 | | 4.601 | <0.001*** |
| Age (24m) | 0.069 | | 0.060 | | 1.157 | 0.255 |
| Condition(IDS):Age(24m) | -0.160 | | 0.063 | | -2.532 | 0.016* |

**Supplementary Table 6. Final model for Minimum F0 (ERB) for the 18-month-old and 24-month-old group**

| ***Parameters*** | | ***Estimate*** | | ***SE*** | ***t-value*** | ***p*** |
| --- | --- | --- | --- | --- | --- | --- |
|  | *Fixed factors* | |  | |  |  |
| (Intercept) | 2.558 | | 0.043 | | 60.138 | <0.001*** |
| Condition (IDS) | 0.067 | | 0.030 | | 2.219 | 0.030* |
| Tone2 | -0.239 | | 0.040 | | -6.159 | <0.001*** |
| Tone3 | -0.365 | | 0.036 | | -10.062 | <0.001*** |
| Tone4 | -0.136 | | 0.033 | | -4.122 | <0.001*** |
| Age (24m) | 0.045 | | 0.051 | | 0.881 | 0.384 |
| Condition(IDS):Age(24m) | -0.112 | | 0.043 | | -2.607 | 0.012* |

**Supplementary Table 7. Model for Maximum F0 (ERB) for the 18-month-old group**

| ***Parameters*** | | ***Estimate*** | | ***SE*** | ***t-value*** | ***p*** |
| --- | --- | --- | --- | --- | --- | --- |
|  | *Fixed factors* | |  | |  |  |
| (Intercept) | 2.626 | | 0.044 | | 59.825 | <0.001*** |
| Condition (IDS) | 0.148 | | 0.043 | | 3.407 | 0.003** |
| Tone2 | -0.009 | | 0.038 | | -0.233 | 0.816 |
| Tone3 | -0.120 | | 0.037 | | -3.224 | 0.001** |
| Tone4 | 0.116 | | 0.035 | | 3.268 | 0.001** |

**Supplementary Table 8. Model for Maximum F0 (ERB) for the 24-month-old group**

| ***Parameters*** | | ***Estimate*** | | ***SE*** | ***t-value*** | ***p*** |
| --- | --- | --- | --- | --- | --- | --- |
|  | *Fixed factors* | |  | |  |  |
| (Intercept) | 2.694 | | 0.040 | | 67.234 | <0.001*** |
| Tone2 | -0.100 | | 0.043 | | -2.250 | 0.025* |
| Tone3 | -0.085 | | 0.042 | | -2.066 | 0.040* |
| Tone4 | 0.130 | | 0.041 | | 2.829 | 0.005** |

**Supplementary Table 9. Model for Minimum F0 (ERB) for the 18-month-old group**

| ***Parameters*** | | ***Estimate*** | | ***SE*** | ***t-value*** | ***p*** |
| --- | --- | --- | --- | --- | --- | --- |
|  | *Fixed factors* | |  | |  |  |
| (Intercept) | 2.537 | | 0.039 | | 64.388 | <0.001*** |
| Condition (IDS) | 0.067 | | 0.028 | | 2.361 | 0.019* |
| Tone2 | -0.224 | | 0.041 | | -5.507 | <0.001*** |
| Tone3 | -0.336 | | 0.040 | | -8.497 | <0.001*** |
| Tone4 | -0.089 | | 0.038 | | -2.352 | 0.019* |

**Supplementary Table 10. Model for Minimum F0 (ERB) for the 24-month-old group**

| ***Parameters*** | | ***Estimate*** | | ***SE*** | ***t-value*** | ***p*** |
| --- | --- | --- | --- | --- | --- | --- |
| (Intercept) | 2.566 | | 0.038 | | 67.497 | <0.001*** |
| Tone2 | -0.214 | | 0.040 | | -5.378 | <0.001*** |
| Tone3 | -0.354 | | 0.039 | | -9.097 | <0.001*** |
| Tone4 | -0.158 | | 0.038 | | -4.109 | <0.001*** |

**Supplementary Table 11. Final model for F0 range (ERB) for the 18-month-old and 24-month-old group**

| ***Parameters*** | ***Estimate*** | ***SE*** | ***t-value*** | ***p*** |
| --- | --- | --- | --- | --- |
|  | *Fixed factors* | | | |
| (Intercept) | 1.130 | 0.740 | 15.293 | <0.001*** |
| Condition (IDS) | 0.107 | 0.046 | 2.325 | 0.020* |
| Tone3 | 0.036 | 0.082 | 0.441 | 0.660 |
| Tone4 | 0.019 | 0.078 | 0.252 | 0.801 |
| Age (24m) | -0.169 | 0.098 | -1.726 | 0.087 |
| Tone3:Age (24m) | 0.246 | 0.115 | 2.139 | 0.033* |
| Tone4:Age (24m) | 0.338 | 0.112 | 3.043 | 0.002** |

## Supplementary Figures

**Supplementary Figure 1.** Box plots of Maximum F0 (ERB) for ADS and IDS addressing 18-month-old and 24-month-old children

**
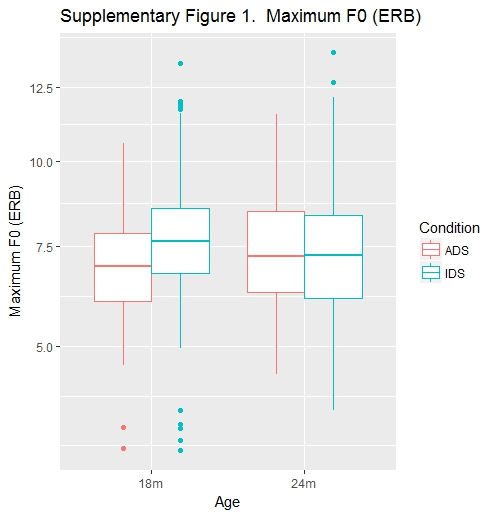
**

**Supplementary Figure 2.** Box plots of Minimum F0 (ERB) for ADS and IDS addressing 18-month-old and 24-month-old children**.**

**
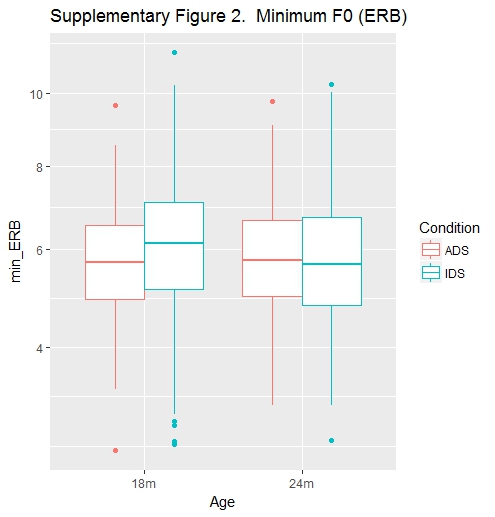
**

**Supplementary Figure 3.** Box plots of F0 range (ERB) for ADS and IDS addressing 18-month-old and 24-month-old children**.**

**
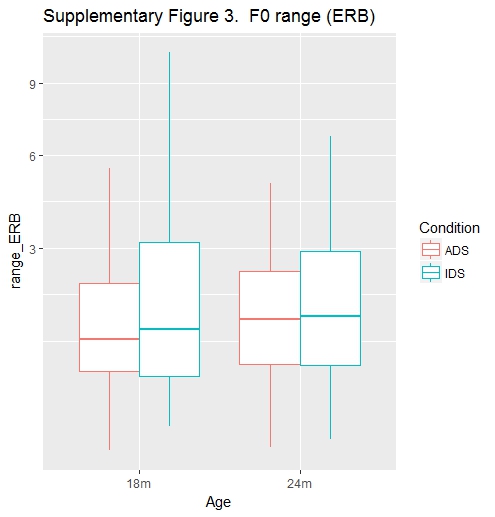
**
